# Caregivers' Experiences Regarding Antibiotic Usage in Treating Children's Upper Respiratory Tract Infections in Southern Tanzania

**DOI:** 10.24248/eahrj.v7i2.731

**Published:** 2023-11-30

**Authors:** Zephania Saitabau Abrahama, Paulo Mahegaa, Aveline Aloyce Kahinga

**Affiliations:** a Department of Surgery, School of Medicine and Dentistry, University of Dodoma, Dodoma, Tanzania

## Abstract

**Introduction:**

Upper respiratory tract infections (URTIs) are common illnesses, especially in children and account globally for a substantial proportion of consultations with family doctors. The objective of this study was to assess knowledge, attitude and practice of caretakers regarding antibiotic usage in treating URTIs in Southern Tanzania

**Methods:**

A cross-sectional hospital-based study was conducted at Iringa Regional Referral Hospital in Southern Tanzania from March to June 2022 involving 300 caregivers. Data was analyzed using Statistical Package for Social Sciences (SPSS) version 23.

**Results:**

About two-thirds (71.7%) of the caregivers had poor knowledge regarding antibiotic usage in treating children's URTIs. In this study, 96.7% of the caregivers correctly identified amoxicillin as the most prescribed medication for treatment of children's URTIs. However, about two-thirds (65.0 %) of the parents were aware of the antibiotic resistance that could be caused by misuse of antibiotics. In this study 50.7% of the respondents had poor attitude while 49.3% had good attitude regarding antibiotic usage in treating children's URTIs. Similarly, 53% of the respondents thought local medications are better than antibiotics in treatment of URTIs. Regarding practices, 75.3% of the respondents used to complete the dose of antibiotics when prescribed for treatment of URTIs and 69.7% used to treat their children at home when having URTIs. A significant association was found between the majority of the socio-demographic characteristics of the respondents and the level of knowledge. Similarly, there was a statistically significant association between knowledge and attitude of caregivers pertaining to antibiotic usage in treating children's URTIs.

**Conclusions:**

There is lack of knowledge among caregivers regarding antibiotic usage for treating children's URTIs at the regional hospital and also more than half of the caregivers had poor attitude towards antibiotic usage. Therefore, caregiver's educational interventions should be implemented through health promotions and educational campaigns that should be mainly conducted by medical doctors since they were reported by the caregivers to be the commonest source of information.

## INTRODUCTION

Upper respiratory tract infections (URTIs) are common illnesses, especially in children and account globally for a substantial proportion of consultations with family doctors. Children usually suffer from 4 to 6 respiratory tract infections per year. The most common etiology is viruses including rhinovirus, influenza virus, adenovirus, enterovirus, and respiratory syncytial virus. Although fewer than 10% of URTI cases are caused by bacteria, the most common bacterial aetiology is *Streptococcus pyogenes* that is a Group A streptococcus.^1^

In pediatric cases of URTIs, prescription of antibiotics is a common practice despite evidence supporting the fact that most infections are caused by viruses. Antibiotic abuse in treatment of URTIs in children is the most common factor leading to antibiotic resistance; thus, the judicious use of antibiotics in children is extremely crucial.^2,3^ Globally, more than 50% of children with URTIs continue to be treated with antibiotics receiving an average of 2 to 3 prescriptions per year.^4^ However, some studies in the United States showed that 43.3% and 78% of parents correctly stated that viruses cause colds and flu, respectively.^1,5^ Also in the same country it was found that the majority of Latino parents (94.5%) knew viruses caused colds and flu and also they agreed that bacteria could be the possible implicated causes.^6^

Several studies conducted in Malaysia have reported URTI to be the most common infection for which antibiotics was prescribed in hospitals and it was as high as 31%–52% in primary care settings.^7,8^

Inappropriate prescription of antibiotics from doctors and poor patients' knowledge regarding antibiotic usage and complication has led to an increase in the misuse of antibiotics.^9^

Caregivers may be contributing to the emerging drug resistance emanating from irrational use of antibiotics. From the available literature, there is no study from Southern Tanzania that aimed at determining whether caretakers were knowledgeable on use of antibiotics in treatment of children's URTIs. The study aimed to address this gap.

## MATERIALS AND METHODS

### Study Design, Area and Study Duration

A hospital based cross-sectional study was conducted at Iringa Regional Referral Hospital from March to June 2022. Iringa Regional Referral Hospital is the largest hospital in the Southern zone of Tanzania and serves as a centre for receiving all the patients referred from other health facilities in the mentioned zone. Iringa Regional Referral Hospital is one of 26 regional hospitals in Tanzania, offering care to a population of over 1.5 million people. Departments include; Outpatient department, Medical, Pediatrics and Child Health, Surgical, Orthopedics and traumatology, Obstetrics and Gynecology, Ophthalmology, Dental, Radiology, and Laboratory Departments.

### Sampling Technique, Sample Size and Study Population

A convenience sampling technique was utilized to recruit three hundred caretakers of children aged 1 month to 12 years upon consenting to participate.

### Inclusion Criteria

All caregivers of children aged 1 month to 12years who consented to participate.

### Exclusion Criteria

Caregivers of children aged 1 month to 12 years who were not mentally fit to consent to participate.

### Data Collection Tools

Questionnaires were semi-structured and translated into the Swahili language to ensure no misinterpretation of questions during interview for maximum reliability.

### Data Analysis

Data was analyzed using Statistical Package for Social Sciences (SPSS) version 23. Where appropriate odd's ratio was calculated to establish any association existing between variables and a p-value <0.05 was considered to be statistically significant.

### Ethical Considerations

Ethical clearance was obtained from the Ethics and Research Committee of the University of Dodoma. The study was approved by the Institutional Research Review Committee (IRREC) of the University of Dodoma on 28^th^ October 2021 under the approval number MA/84/261/02/A'. The permission to carry this study was obtained from the executive director of the Iringa regional hospital. All prospective participants gave a written informed consent before they were recruited. Data that was collected from this study was kept confidential since no names of the study participants appeared in the questionnaires.

## RESULTS

### Socio-Demographic Characteristics of the Respondents

In this study, a total of 300 respondents were recruited where majority were from urban area, 245 (81.7%) while those from rural area were 55 (18.3%) respondents. Females, 219 (73%) predominated in this study and males were 81 (27%). Majority of study participants belonged to the age group, 28–37 years (50.3%) and the least number of participants were aged ≥48 years, 15 (5.1%). Regarding marital status, majority of the respondents were married, 180 (60%) while 9 (3%) were divorced. In terms of level of education, most respondents had secondary level education, 122 (40.7%) and 26 (8.7%) had non-formal education. Similarly, 141 (47%) study participants were self-employed while 51 (17%) were salaried/employed (Table 1).

**TABLE 1:** Socio-demographic Characteristics of the Respondents, (N = 300)

Socio-demographic characteristics	Sub variable	Frequency (%)
Gender	Male	81 (27.0)
	Female	219 (73.0)
Age (years)	18–27	97 (32.3)
	28–37	151 (50.3)
	38–47	37 (12.3)
	≥48	15 (5.1)
Marital status	Married,	184 (61.3)
	Widowed	32 (10.7)
	Divorced	8 (2.7)
	Cohabiting	41 (13.7)
	Not married	35 (11.7)
Relation to the child	Mother	180 (60.0)
	Father	76 (25.3)
	Caretaker	42 (14.0)
	Other	2 (0.7)
Educational level	Non-formal	26 (8.7)
	Primary	59 (19.7)
	Secondary,	122 (40.7)
	Certificate/Diploma	58 (19.3)
	Degree/Masters	35 (11.7)
Occupation	Student	16 (5.3)
	Housewife	60 (20.0)
	Self-employed,	141 (47.0)
	Salaried/employed	51 (17.0)
	Unemployed	32 (10.7)
Residence	Urban area	245 (81.7)
	Rural area	55 (18.3)

### Knowledge of Respondents Regarding Antibiotics Usage in Treating Upper Respiratory Tract Infections

Majority of the respondents, 209 (96.7%) knew what antibiotics were and 107 (35.7%) respondents knew viruses to be the causative agent of URTIs. Similarly, 285 (95%) study participants mentioned amoxicillin to be the most commonly prescribed antibiotic in treatment of URTIs and 261 (87%) respondents reported that children with flu-like symptoms get better faster when antibiotics are given. In the same study, 276 (92%) respondents reported antibiotics could prevent complications from URTIs and 230 (76.7%) respondents knew that inappropriate use of antibiotics reduced their efficacy and may lead to bacterial resistance. Generally, in this study majority of the respondents (71.7%) had poor knowledge while 28.3% had good knowledge regarding antibiotics usage in treating URTIs (Table 2).

**TABLE 2:** Knowledge of Respondents Regarding Antibiotics Usage in Treating Upper Respiratory Tract Infections, (N=300)

Variable	Sub variable	Frequency (%)
What are the symptoms of URTIs?		
	Sore throat	95 (31.7)
	Runny nose	253 (84.3)
	Nasal congestion	169 (56.3)
	Low-grade fever	199 (66.3)
	Facial pressure	49 (16.3)
	Sneezing	79 (26.3)
	Malaise	146 (48.7)
	Myalgia	40 (13.3)
	Others	3 (1.0)
What is the causative agent of URTIs?	Bacteria	108 (36)
	Virus	107 (35.7)
	Not sure	85 (28.3)
Do you know what Antibiotics are?	Yes	209 (96.7)
	No	4 (1.3)
	Not sure	6 (2.0)
Which of the following is the most commonly prescribed antibiotic in treatment of URTIs?		
	Amoxicillin	285 (95)
	Azithromycin	11 (3.7)
	Others	4 (1.3)
Do antibiotics prevent complications from upper respiratory tract infections?		
	Yes	276 (92)
	No	8 (2.7)
	Not sure	16 (5.3)
Children with flu-like symptoms get better faster when antibiotics are given?		
	Yes	261 (87.0)
	No	15 (5.0)
	Not sure	24 (8.0)
As most of the upper respiratory tract infections(e. g. Cold, flu, sore throat, ear infections) are of viral origin, antibiotics should not be given because they are self-limited?		
	Yes	27 (9.0)
	No	144 (48.0)
	Not sure	129 (43.0)
Inappropriate use of antibiotics reduces their efficacy and leads to bacterial resistance?		
	Yes	230 (76.7)
	No	8 (2.7)
	Not sure	62 (20.7)
Resistance to antibiotics is a worldwide problem?	Yes	195 (65.0)
	No	16 (5.3)
	Not sure	89 (29.7)
Source of information regarding antibiotic usage	Doctors	259 (86.3)
	Internet	160 (53.3)
	Friends	97 (32.3)
	Others	5 (1.7)
Overall knowledge	Poor Knowledge	215 (71.7)
	Good knowledge	85 (28.3)

### Attitudes of Respondents Regarding Antibiotics Usage in Treatment Of Children's URTIs

In this study, 102 (34%) respondents thought antibiotics could treat bacterial, viral and fungal infections and 182 (60.7%) respondents thought antibiotics could cause allergic reactions as one of their side effects. Similarly, 234 (78%) respondents have ever heard of antibiotic resistance and 159 (53%) disagreed with the statement that local medications are better than antibiotics in treatment of URTIs. Generally, in this study 50.7% of the respondents had poor attitude while 49.3% had good attitude regarding antibiotics usage in treating URTIs (Table 3).

**TABLE 3:** Attitudes of Respondents Regarding Antibiotics Usage in Treatment of Children's URTIs, (N=300)

Variable	Sub variable	Frequency (%)
What do you think antibiotics treat?	Bacterial infections	94 (31.3)
	Viral infections	48 (16.0)
	Fungal infections	22 (7.3)
	All of the above	102 (34.0)
	I don't know	34 (11.3)
Do you think antibiotics have side effects or an allergic reaction?	I agree	182 (60.7)
	I disagree	17 (5.7)
	Not sure	101 (33.7)
Have you heard of antibiotics resistance?	Yes	234 (78.0)
	No	66 (22.0)
Do you think using local medications is better than using antibiotics in treatment of URTIs?		
	I agree	103 (34.3)
	I disagree	159 (53.0)
	Not sure	38 (12.7)
Do you think antibiotics are generally safe?	I agree	188 (62.7)
	I disagree	47 (15.7)
	Not sure	65 (21.7)
Overall Attitude	Poor Attitude	152 (50.7)
	Good Attitude	148 (49.3)

### Practices of Respondents Regarding Antibiotic Usage in Treatment of Children's URTIs

The study has found 226 (75.3%) respondents to have always completed the course of treatment with antibiotics when their children feel better and 270 (90%) respondents reported to seek consultation from doctors for prescription in treatment of URTIs. Similarly, 209 (69.7%) respondents reported to treat children at home when having URTIs and 191 (63.75) reported to have been given any medication by friends and relatives (Table 4).

**TABLE 4:** Practices of Respondents Regarding Antibiotic Usage in Treatment of Children's URTIs, (N = 300)

Variable	Sub variable	Frequency (%)
Do you always complete the course of treatment with antibiotics to your children even if he/she feel better?		
	Yes	226 (75.3)
	No	274 (24.7)
Do you seek a prescription from the doctor for URTIs in children?	Yes	2270 (90.0)
	No	230 (10.0)
Do you keep medications at home?	Yes	2244 (81.3)
	No	256 (18.7)
Do you treat your child at home when having URTI?	Yes	2209 (69.7)
	No	291 (30.3)
Have you ever been given any medication by friends and relatives?	Yes	2191 (63.7)
	No	2109 (36.3)

### Factors that Influence Caregivers to Use Antibiotics in Treating Children's URTIs

The study has found 115 (38.3%) respondents to have compliance with medications when their children were prescribed and 263 (87.7%) respondents sought health care from a pharmacy that is located less than 5 kilometres from their place of residence (Table 5).

**TABLE 5:** Factors That Influence Caregivers to Use Antibiotics in Treating Children's URTIs, (n = 300)

Variable	Sub variable	Frequency (%)
Do you have any compliance in getting medication for your child?	Yes	115 (38.3)
	No	185 (61.7)
Where do you seek for health care for your child when suffering from URTIs?		
	Hospital	179 (59.7)
	Pharmacy	117 (39.0)
	Nowhere/home	3 (1.0)
	Traditional healers	1 (0.3)
How far is the health care facility from your place of residence?	Less than 5km	263 (87.7)
	More than 5km	37 (12.3)

### Association Between Socio-Demographic Characteristics of Respondents and Overall Knowledge Regarding Antibiotics Usage in Treating Children's URTIs

This study has found a significant association between the overall knowledge on antibiotic usage and some socio-demographic characteristics of the study participants like gender, relationship with the child's caretaker, level of education and employment status (their corresponding p-values are less than 0.05) though no association was found between overall knowledge and marital status (p value=0.509) and place of residence (p value=0.064) (Table 6).

**TABLE 6:** Association Between Socio-demographic Characteristics of Respondents and Overall Knowledge Regarding Antibiotics Usage in Treating Children's URTIs, (n =300)

Variable	Sub-variables	Overall Knowledge		P value	Prevalence Odd's Ratio (POR)	95% Confidence Interval
		Poor, n (%)	Good n (%)			
Gender	Female	49 (60.5)	32 (39.5)	.009	0.489	0.284–0.841
	Male	166 (75.8)	53 (24.2)			
Child's caretaker	Mother	134 (74.4)	46 (25.6)	.01		0.042–0.052
	Father	44 (57.9)	32 (42.1)			
	Caretaker	35 (83.3)	7 (16.7)			
	Other	2 (100)	0 (0)			
Marital status	Married	128 (69.6)	56 (30.4)	509		0.394–0.420
Widowed	21 (65.6)	11 (34.4)				
	Divorced	7 (87.5)	1 (12.5)			
	Cohabiting	32 (78.0)	9 (22.0)			
	Not married	27 (77.1)	8 (22.9)			
Level of education	Non-formal	21 (80.8)	5 (19.2)	.000		0.000–0.001
	Primary	53 (89.8)	6 (10.2)			
	Secondary	88 (72.1)	34 (27.9)			
	Certificate/Diploma	38 (65.5)	20 (34.5)			
	Degree/Masters	15 (42.9)	20 (57.1)			
Occupation	Student	12 (75.0)	4 (25.0)	.01		0.000–0.002
	Housewife	47 (78.3)	13 (21.7)			
	Self-employed	105 (73.4)	38 (26.6)			
	Salaried/employed	25 (51.0)	24 (49.0)			
	Unemployed	26 (81.3)	6 (18.8)			
Residence	Urban	170 (69.4)	75 (30.6)	.064	0.541	0.241–1.053
	Rural	45 (81.8)	10 (18.2)			
Total		215 (71.7)	85 (28.3)			

### Association Between Socio-Demographic Characteristics of Respondents and Attitude Level Regarding Antibiotics Usage in Treating children's URTIs

The study has found a statistically significant association between overall attitude and all the mentioned socio-demographic characteristics of the study participants (like gender, child's caretaker, marital status, level of education and employment status and place of residence) since all their corresponding p-values are less than 0.05 (Table 7)

**TABLE 7:** Association Between Socio-demographic Characteristics of Respondents and Overall Attitude Regarding Antibiotics Usage in Treating Children's URTIs, (N =300)

Variable	Sub-variables	Overall Attitude		P value	Prevalence Odd's Ratio (POR)	95% Confidence Interval n (%)
		Poor n (%)	Good n (%)			
Gender	Female	124 (56.6)	95 (43.4)	.001	0.405	0.238 – 0.688
	Male	28 (34.6)	53 (65.4)			
Child's caretaker	Mother	97 (53.9)	83 (46.1)	.003		0.000–0.002
	Father	27 (35.5)	49 (64.5)			
	Caretaker	28 (66.7)	14 (33.3)			
	Other	0 (0.0)	2 (100)			
Marital status	Married	79 (42.9)	105 (57.1)	.017		0.025–0.034
	Widowed	21 (65.6)	11 (34.4)			
	Divorced	5 (62.5)	3 (37.5)			
	Cohabiting	27 (77.1)	14 (34.1)			
	Not married	20 (57.1)	15 (42.9)			
Level of education	Non-formal	21 (80.8)	5 (19.2)	000		0.000–0.000
	Primary	41 (69.5)	18 (30.5)			
	Secondary	55 (45.1)	67 (54.9)			
	Certificate/Diploma	24 (41.4)	34 (58.6)			
	Degree/Masters	11 (31.4)	24 (68.6)			
Occupation	Student	7 (43.8)	9 (56.3)	.000		0.000–0.000
	Housewife	35 (58.3)	25 (41.7)			
	Self-employed	76 (53.1)	67 (46.9)			
	Salaried/employed	11 (22.4)	38 (77.6)			
	Unemployed	23 (71.9)	9 (28.1)			
Residence	Urban	114 (46.5)	131 (53.5)	.02	0.389	0.208–0.727
	Rural	38 (69.1)	17 (30.9)			
Total		215 (71.7)	85 (28.3)			

### Association Between Overall Knowledge and Attitude Regarding Antibiotics Usage in Treating Children's URTIs

In this study, there is a statistically significant association between overall knowledge and attitude regarding antibiotics usage in treating children's URTIs since the p-value=0.000 (Table 8).

**TABLE 8:** Association Between Overall Knowledge and Attitude Regarding Antibiotics Usage in Treating Children's URTIs, (n =300)

Overall Attitude	Overall Knowledge		P value
	Poor, n (%)	Good, n (%)	
Poor Attitude	126 (82.9)	26 (17.1)	.000
Good Attitude	89 (60.1)	59 (39.9)	
Total	215 (71.7)	85 (28.3)	

### Association Between Overall Knowledge and Practices Regarding Antibiotics Usage in Treating Children's URTIs

There is a statistically significant association between overall knowledge and practices regarding antibiotics usage in treating children's URTIs since the p-value=0.000 (Table 9).

**TABLE 9:** Association Between Overall Knowledge and Practices Regarding Antibiotics Usage in Treating Children's URTIs (n = 300)

Overall Practices	Overall Knowledge		P value
	Poor, n (%)	Good, n (%)	
Poor Practices	126 (82.9)	26 (17.1)	.000
Good Practices	89 (60.1)	59 (39.9)	
Total	215 (71.7)	85 (28.3)	

## DISCUSSION

Antibiotic abuse in URTIs in children is the most common factor leading to antibiotic resistance; thus, judicious use of antibiotics among children is extremely crucial and to the best of our knowledge this is the first study in Tanzania to explore knowledge, attitude and practice of caregivers regarding antibiotic usage in treating children's URTIs.

This study has found that 71.7% of parents/caretakers had poor knowledge regarding antibiotics usage in treating children with URTIs. Interestingly, 95% of the respondents recognized amoxicillin as one of the antibiotics commonly prescribed for treating children's URTIs and 35.7% were aware that URTIs are mainly viral in origin. These findings are comparable to those reported from Saudi Arabia and Macedonia which reported that 38.5% and 24.2%, respectively recognized the correct antibiotics for treating URTIs.^10,11^ This may be due to lack of education between bacterial and viral infections and on top of that this finding should be an alarm since inappropriate use of antibiotics to treat URTIs leads to antibiotic resistance.

In this study, 60.7% of the respondents acknowledged antibiotics to have side effects that is much lower than the finding reported from the study that was conducted in Cyprus that reported 93% of the respondents knew antibiotics to have side effects.^12^

Regarding source of antibiotics usage in treating URTIs, this study has found doctors (86.3%) to be the commonest source of information, a finding that correlates with what has been reported in Saudi Arabia and Cyprus.^12,13^ Doctors should be trained on communication since they are the commonest source of information in this study.

Pertaining the level of education of the caretakers, this study has found a significant association between the overall knowledge on antibiotics usage and some socio-demographic characteristics of the study participants like gender, relationship with the child's caretaker, level of education and employment status though no association was found between overall knowledge and marital status and place of residence. These results are comparable to those obtained from similar studies conducted in Cyprus, Macedonia as well as Malaysia.^10,12,14^

Regarding attitudes of caregivers on antibiotics usage in treating URTIs, 49.3% had good attitude towards use of antibiotics in treating such URTIs. However, some attitudes need to be corrected for example majority of caretakers used to keep medications at home and treat their children at home and others need to be recommended positively like the tendency of caregivers to seek prescription from doctors for treatment of URTIs in children and their act of completing the course of treatment with antibiotics when they feel better. Such findings appear to be higher than what has been reported from the study that was conducted in Malaysia.^15^

In terms of association between knowledge and attitudes towards antibiotics usage in treating URTIs in children, the study has found a statistically significant association between overall knowledge and attitude regarding antibiotics usage in treating children's URTIs. Such findings appear to be similar to what has been reported in the study that was done in Malaysia in 2017.^16^

In this study, 34% reported antibiotics could treat bacterial, viral and fungal infections and more than three quarters (78%) have ever heard of antibiotic resistance. Similarly, 62.7% of the respondents reported antibiotics to be generally safe when used and 60.7% thought antibiotics had side effects or allergic reactions Such findings are similar to those from Malaysia where most parents had a misconception that most of the cold and fever are responsive to antibiotics and can cure them.^3^

Pertaining the association between socio-demographic characteristics of respondents and attitude level regarding antibiotics usage in treating children's URTIs, the study has found a statistically significant association between overall attitude and all the mentioned socio-demographic characteristics of the study participants (like gender, child's caretaker, marital status, level of education, employment status and place of residence). The findings are in line with what has been found in Jordan where the mother's educational level had a strong association with attitude on antibiotic usage.^17^

Regarding caregivers' practices on antibiotic usage in treating URTIs, the study has found 90% of the respondents to have sought prescription from doctors when their children are suffering from URTIs and also 75.3% of them used to complete the course of treatment with antibiotics to their sick children even if they feel better. These findings correlate with what has been reported in Palestine where 76.6% of the parents used to follow pediatricians' directives when prescribed medications,^18^ but dissimilar to what was found in Malaysia where most parents did not perceive that doctors would prescribe an antibiotic for URTIs.^16^

In terms of association between knowledge and practices on antibiotic usage regarding treatment of children's URTIs, there is a statistically significant association between overall knowledge and practices regarding antibiotics usage in treating children's URTIs (*P* value=.000). The finding correlate with what was previously reported in Tanzania.^19^

Pertaining factors that tend to influence antibiotic usage among caregivers in treating children's URTIs, the study has found majority of the respondents (61.7%) to have no compliance with the prescribed medications and also majority (87.7%) travels less than 5km from their home place to nearby health facility to seek health services. Their tendency of non-compliance is alarming since it may add to the ongoing antibiotic resistance. These findings correlate with those previously reported from Malaysia, Tanzania, and Mongolia.^14,19,20^

On top of that, the study has found association between overall knowledge and practices regarding antibiotics usage in treating children's URTIs.

## Study Limitation

The study was conducted at a single regional referral hospital in Southern Tanzania and involving a modest study population and therefore results cannot be generalized countrywide.

## CONCLUSION

There is lack of knowledge among caregivers regarding antibiotics usage for treating children's URTIs at Iringa Regional Referral Hospital and also more than half of the caregivers had poor attitude towards antibiotics usage. Therefore, caregivers' educational interventions should be implemented though health promotions and educational campaigns that should be mainly conducted by medical doctors since they were reported to be the commonest source of information on antibiotics usage.
